# Production of Graft Copolymers of Cod Collagen with Butyl Acrylate and Vinyl Butyl Ether in the Presence of Triethylborane—Prospects for Use in Regenerative Medicine

**DOI:** 10.3390/polym15153159

**Published:** 2023-07-25

**Authors:** Lyudmila Semenycheva, Victoria O. Chasova, Nikita L. Pegeev, Marina A. Uromicheva, Alexander V. Mitin, Yulia L. Kuznetsova, Ekaterina A. Farafontova, Yulia P. Rubtsova, Daria D. Linkova, Marfa N. Egorikhina

**Affiliations:** 1 Faculty of Chemistry, National Research Lobachevsky State University of Nizhny Novgorod, 23, Gagarin Ave., 603022 Nizhny Novgorod, Russia; tchasowa.vika@yandex.ru (V.O.C.); nikita.pegeev@mail.ru (N.L.P.); kozinamarina1@yandex.ru (M.A.U.); ckp@ichem.unn.ru (A.V.M.); kyul@yandex.ru (Y.L.K.); 2 Federal State Budgetary Educational Institution of Higher Education, Privolzhsky Research Medical University of the Ministry of Health of the Russian Federation, 603005 Nizhny Novgorod, Russia; ekaterina_farafontova@mail.ru (E.A.F.); rubincherry@yandex.ru (Y.P.R.); linckovadaria@yandex.ru (D.D.L.); egorihina.marfa@yandex.ru (M.N.E.)

**Keywords:** graft copolymer, cod collagen, vinyl butyl ether, butyl acrylate, triethylborane, alternating copolymer, scaffold, (co)polymerization, methods of physical and chemical analysis, cytotoxicity

## Abstract

Collagen is a suitable material for regenerative medicine because it is characterized by its good biocompatibility. However, due to its fibrillar structure, it cannot organize itself into three-dimensional porous structures without additional modification. The introduction of synthetic monomer elements into the collagen macromolecules is a technique used to form three-dimensional, collagen-based, branched, and crosslinked structures. New types of graft copolymers made from cod collagen with a butyl acrylate and vinyl butyl ether copolymer in aqueous dispersion were obtained in the presence of triethylborane by a radical mechanism. The process of graft copolymer formation proceeded as usual by radical initiation, through radicals formed during triethylborane oxidation by oxygen residues, collagen borination, and reversible inhibition with the participation of a boroxyl radical. The characteristics of the graft copolymers were determined using methods of physical and chemical analysis (GPC, SEM, IR spectroscopy, etc.), while the cytotoxicity was assessed using the MTT assay method. It is shown that the grafting of alternating blocks of butyl acrylate and vinyl butyl ether to the protein macromolecules results in changes in the morphological pattern of the graft co-polymer in comparison with native collagen. This is manifested in the development of consolidations around the collagen fibers of the structural matrices, with the co-polymer cellular structure consisting of interpenetrating pores of unequal size. Additionally, it is important that the graft co-polymer solutions are not toxic at a certain concentration. The above properties confirm the promising nature of the technique’s application as the basis for producing new materials for regenerative medicine.

## 1. Introduction

New materials for regenerative medicine, namely, scaffolds able to act as cell carriers that allow the cells to maintain their functional activity, should also undergo bioresorption during the process of repair of the damaged tissues, but their biocompatibility and low cytotoxicity are of paramount importance [1,2,3]. In this respect, marine collagen isolated from fish skin has special properties. Unlike animal collagen, it has higher values of antimicrobial and antioxidant activity, while being 96% similar to human collagen in its amino acid composition and sequence of amino acid links. Furthermore, there is no risk of it carrying anthropozoonotic diseases to humans, unlike animal collagen, while its use is also without religious restrictions [4,5,6,7,8,9]. In fact, there is particularly relevant research related to the possibility of using fish waste products, such as fish skin, to solve production problems [4,5,6,7,8,9]. All the above aspects make the use of marine collagen in regenerative medicine a promising area, attracting the research interest of many scientists [1,2,3,4,5,6,7,8,9].

However, collagen, with all its advantages, has a disadvantage: its fibrillar coplanar structure. Thus, it has no ability to self-organize to form three-dimensional porous structures, thus restricting its use in regenerative medicine as scaffolds if unmodified. Any collagen used in regenerative medicine is most often incorporated as a part of composite materials [2,10,11,12,13]. Different ways to obtain three-dimensional structures using collagen/gelatin have been proposed, for example, obtaining composites based on collagen with grafted synthetic fragments modifying its three-dimensional structure [5,13,14,15,16,17,18,19,20,21,22,23,24,25]. One of the methods for obtaining such copolymers is graft copolymerization, used to introduce the modifying additives [5,14,15,16,17,18,19,26]. Investigation of the graft polymerization of different synthetic fragments onto collagen and any peculiarities related to the nature of the synthetic monomers used is an important step in the creation of new materials for regenerative medicine.

The use of trialkylboranes in the graft polymerization of vinyl monomers onto the surface of natural polymers [18,23,24,27,28,29,30] is described in the literature. An important feature of organoboranes is the possibility of low-temperature polymerization, their participation in chain transfer reactions, and their ability to boride collagen, thus affecting the structure of the resulting copolymers. The extensive possibilities of alkylboranes as radical (co)polymerization reagents allow consideration of them as promising compounds for the investigation of their possibilities in creating new macromolecular designs. In this regard, a triethylborane-hexamethylenediamine (TEB-HMDA) complex, releasing triethylborane (TEB) in an acid medium, was used in this study to initiate such grafting onto collagen.

In contrast to earlier studies [18,23,24,27,28,29,30] and in order to obtain a collagen copolymer with an original structure and properties, a mixture of butyl acrylate (BA) and vinyl butyl ether (VBE) was used instead of just a single synthetic monomer. In an excess of VBE, the radical copolymerization of the BA-VBE pair produced a predominantly alternating copolymer. The cause of this was that VBE produces not a homopolymer at radical initiation conditions, but copolymerizes with vinyl series monomers [31]. In copolymerization with BA in an excess of VBE, and due to the high radical activity of BA, a composite, homogeneous chain is formed from BA and VBE links [32]. Introduction of VBE links into the macromolecular chain of the copolymer with collagen is interesting, as the synthetic copolymer BA-VBE has the advantage of high mechanical stability as compared with polybutylacrylate (PBA). In addition, we can count on its good biocompatibility, as VBE polymers are known to be medicinal healing agents [33].

The main purpose of our studies was to obtain a new composite material based on a biodegradable copolymer of cod collagen (CC) with BA and VBE (CC-PBA-PVBE), to characterize its properties using methods of physical and chemical analysis, and to conduct enzymatic hydrolysis of the resulting graft copolymer to determine the structure of the copolymer’s synthetic fragment.

The main objectives were as follows:

- To adapt previously determined conditions for obtaining graft copolymers of collagen with methyl methacrylate (MMA) and acrylamide (AA) [18] using copolymer BA-VBE grafting;
- To characterize the target product using methods of physical and chemical analysis: gel permeation chromatography (GPC), scanning electron microscopy (SEM), elemental analysis (CHNS), and IR spectroscopy;
- To hydrolyze the CC-PBA-PVBE graft copolymer using collagenase, to isolate the grafted synthetic fragment, and to analyze it using GPC and Fourier-transform infrared spectroscopy (FTIR);
- To evaluate the cytotoxicity of the grafted copolymer using the MTT assay method.

## 2. Materials and Methods

### 2.1. Materials

To obtain the graft copolymers, commercial reagents were used during this study: firstly, the polymerization inhibitor (hydroquinone) was removed from the BA through repeated washing of the monomer with 10% sodium hydroxide solution and water to a neutral medium; then, the remaining moisture was removed by drying the BA for 24 h over calcium chloride. After all these manipulations, the purified monomer was distilled under vacuum. Non-purified VBE was used as the second monomer.

Marine collagen isolated from cod skin tissues using extraction in 3% acetic acid for one day according to the method in [34] was chosen as the natural polymer onto which the synthetic PBA-PVBE copolymer was grafted.

### 2.2. Synthesis of the Graft Copolymer CC-PBA-PVBE

**Scheme 1:**
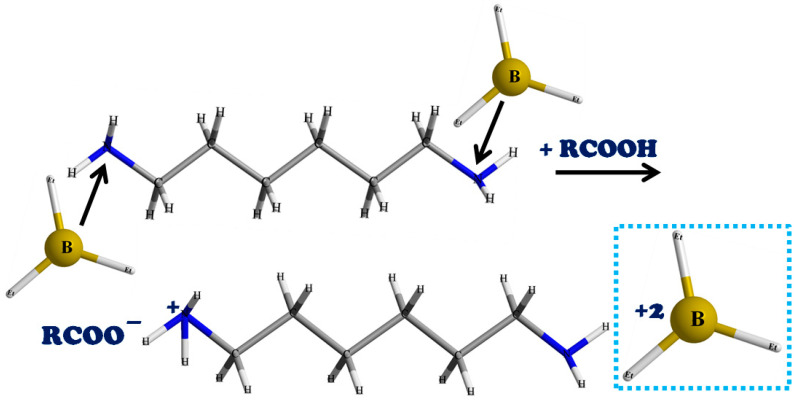
TEB release when acid is added.

The graft copolymer was synthesized in an aqueous collagen/monomers emulsion at room temperature in an argon stream at a mass ratio CC:BA:VBE = 2.5:1:10 (10% collagen mass fraction in the aqueous emulsion). The aqueous dispersion of collagen with BA was a separate barbotage with argon for 15 min at room temperature with low-speed stirring (300 rpm), then the VBE was added. TEB used as a polymerization initiator was introduced into the reaction mixture as a non-oxidizable compound, TEB-HMDA complex, which was destroyed by catalytic amounts of methacrylic acid (see Scheme 1), which previously purified by crystallization. The resulting mixture was stirred with a magnetic stirrer (800 rpm) for 5 h in an argon flow. With the reaction completed, the mixture was separated into aqueous and organic phases.

### 2.3. Enzymatic Hydrolysis

To isolate and analyze the synthetic fragment of the graft copolymer, it was subjected to enzymatic destruction by collagenase, which is a specific proteolytic enzyme breaking peptide bonds in natural collagen down to oligomers and amino acids. The graft copolymer was neutralized to pH 7.2–7.4, and collagenase was added at a collagen:enzyme mass ratio of 50:1 and left for 3 days. Then, the resulting mixture was filtered through a paper filter, which was washed after filtration either in chloroform or in tetrahydrofuran, before further study of the polymer.

### 2.4. Gel-Penetrating Chromatography

The molecular weight characteristics of the aqueous solutions were determined by GPC using a Shimadzu CTO20A/20AC high-performance liquid chromatograph (Shimadzu, Kyoto, Japan) with an LC-Solutions-GPC software module; separation was performed using a Tosoh Bioscience TSK gel G3000SWxl column with 5 µm diameter pores; an ELSD-LT II low-temperature light scattering unit was used as a detector; and 0.5 M acetic acid solution at a 0.8 mL/min flow rate was used as the eluent, while narrow-dispersed dextran samples (Fluca) of 1–410 kDa molecular weight were used for calibration.

The synthetic polymer isolated by enzymatic hydrolysis from the copolymer was analyzed using a Prominence LC-20VP system (Shimadzu, Kyoto, Japan) under the following conditions: Tosoh Bioscience column (polystyrene-divinylbenzene gel, 106 and 105 Å pore size) (Tosoh, Tokyo, Japan); 40 °C column temperature; tetrahydrofuran eluent at 1 mL/min flow rate. The detectors were a differential refractometer and a UV detector (λ = 254 nm). Narrowly separated PMMA standards were used for calibration.

### 2.5. FTIR Spectroscopy

An IRPrestige-21 spectrophotometer (Shimadzu, Kyoto, Japan) was used to record the absorption spectra—4000–550 cm^−1^ wave number range, ≤±0.05 cm^−1^ error. The polymer films had been prepared on a KBr reflection plate.

**Figure 1:**
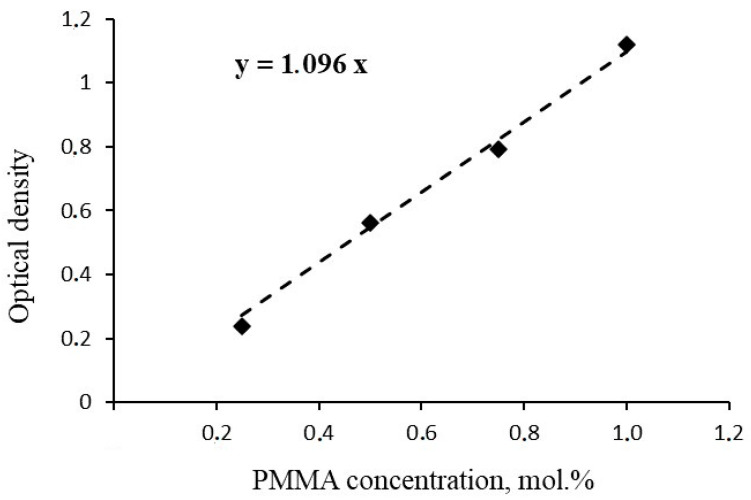
Calibration graph to determine copolymer composition using FTIR spectroscopy with alkyl methacrylate.

Three repeated samples were used to determine the synthetic polymer composition. An accurately weighed quantity of the polymer was dissolved in 5 mL of chloroform and placed in 0.26 mm optical path KBr cuvettes for its IR spectra to be recorded using a Shimadzu FTIR-8400S FTIR spectrophotometer. The wave number range was 5500–550 cm^−1^ and the determination error ≤ ±0.05 cm^−1^. The band at 1726 cm^−1^ for the carbonyl group (acrylate fraction) was chosen as the analytical band. Optical density values (D) were received from the IR spectra of the analyzed samples. The copolymer composition was determined using a calibration graph, based on the characteristic peak area. The calibration graph with coordinates of absorption intensity (optical density) vs. concentration (Figure 1) was built using the absorbance of polymethylmethacrylate (PMMA) solutions. To build it, we integrated spectra of PMMA solutions in chloroform with four exactly known concentrations (0.25–1% PMMA). The absorption intensity was determined using the “baseline” method [35]. The error in determining the concentration of the fragments in the copolymer was ±5%.

The equation of the calibration curve line was applied to determine the PMMA concentration corresponding to the obtained optical density:

(1) y=1.096×x

where *y* is the optical density (*D*), *x* is the PMMA concentration determined using the IR-spectrum (*C*_1_).

Then, the mass fraction (ɷ*_BA_*_,%_) was defined and used as the basis for the mole fraction (χ*_BA_*_,%_) of butyl acrylate determination using Formulas (2) and (3), respectively:

(2) $$ɷBA,\%=\frac{M1}{M2}\times \frac{C1}{C2}$$

(3) $$\chi BA,\%=\frac{ɷBA,\%*M3}{ɷBA,\%\times M3+M1\times (100\%-ɷBA,\%)}$$

where *M*_1_ is the molar mass of *BA*, *M*_2_ is the molar mass of MMA, *M*_3_ is the molar mass of VBE, *C*_1_ is the concentration of the PMMA specified using the IR spectrum, and *C*_2_ is the concentration of the analyzed sample of the PBA-PVBE copolymer.

### 2.6. Scanning Electron Microscopy

The surface of the lyophilically dried, grafted CC-PBA-PVBE copolymer was studied using a JSM-IT300 scanning electron microscope (SEM) (JEOL Ltd., Tokyo, Japan) with a ≤5 nm diameter electron probe (operating voltage 20 kV), using low-energy secondary electron and back-scattered electron detectors in low vacuum mode to eliminate any charge from the samples.

### 2.7. Elemental Analysis

The sample was analyzed for elemental composition after liquid phase evaporation and bringing it to a constant weight. Samples were analyzed by CHNS-analysis on a vario EL cube elemental analyzer for the simultaneous determination of CHNS(O).

### 2.8. Cytotoxicity Examination via MTT Assay

Cytotoxicity was evaluated using the MTT assay method. The studied film samples, once brought to a constant weight in a vacuum oven, were placed in a prepared growth medium (DMEM/F12 containing dissolved antibiotics and 2% calf fetal serum). These samples and the growth medium were placed in a CO_2_ incubator for 24 h at standard conditions (37 °C, 5% CO_2_). After 24 h of incubation, an extract was taken from the test samples, and a series of dilutions of each extract with growth medium was prepared at ratios of 1:1, 1:2, 1:4, and 1:8. To examine the samples in the separated aqueous emulsion phase, each sample solution was taken as the undiluted extract.

Human dermal fibroblasts (HDFs) of 4–6 passages were used to study the samples with an active, morphologically homogeneous culture well adhering to the plastic plate being used. The culture cells’ immunophenotype corresponded to that of mesenchymal cells, and the culture viability was >97%. The HDFs were seeded in the wells of a 96-well flat-bottomed plate at 10,000/cm^2^ density. Growth medium DMEM/F12 with antibiotics (100 units/mL penicillin, 100 μg/mL streptomycin) and 10% fetal calf serum was used for the cultivation (standard conditions, 24 h). After 24 h, uniform growth of typical fibroblast-like cells as a subconfluent monolayer was recorded in all wells. After 24 h of cultivation, the growth medium above the cells was replaced with the test solutions.

Different concentrations of the extract were added to the HDF cultures in the 96-well plate. Eight wells were assigned for each concentration. The plate with samples was placed into a CO_2_ incubator for 72 h. After this time, changes in cell morphology under the influence of the extract and its dilutions were recorded (light microscopy, Leica DM IL LED biological inverted microscope with accessories, Leica Mycrosystems Ltd., Wetzlar, Germany; LAS V4.13 software).

After photodocumentation, MTT solution (20 μL) was added to each well, and the cells were incubated with the MTT for 3 h in a CO_2_ incubator. (The standard concentration 5 mg/mL of MTT solution in phosphate buffer was used.) After 3 h of incubation, the supernatant was taken. Then, an equal volume of DMSO solution was substituted, and the optical density (*OD*) at 540 nm was recorded using a Sunrise tablet reader (Austria).

The relative growth rate (RGR) was calculated according to Formula (4):

(4) $$\mathrm{RGR}\;\left(\%\right)=\frac{average\;OD\;in\;test\;culture}{average\;OD\;in\;control}\times 100$$

Cytotoxicity severity was rated: grade 0 (100% RGR) and grade 1 (75–99% RGR) were taken as showing no cytotoxicity; grade 2 (50–74% RGR) corresponded to mild cytotoxicity; grade 3 (25–49% RGR) corresponded to medium cytotoxicity; grade 4 (0–24% RGR) corresponded to high cytotoxicity [36].

## 3. Results and Discussion

**Scheme 2:**
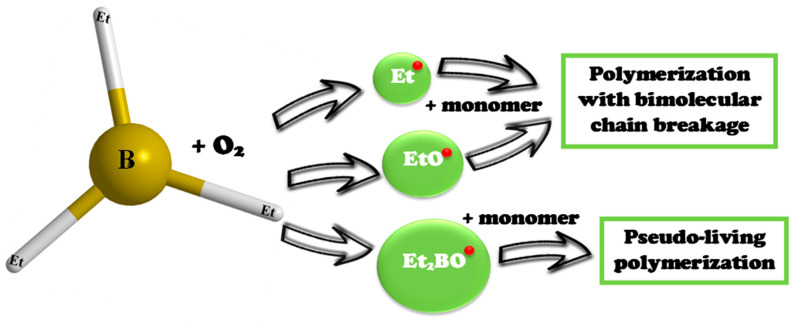
Oxidation reactions of TEB.

Collagen graft copolymers with vinyl monomers formed under the selected process conditions may be produced through several radical reactions, as has previously been noted in the introduction. As in the case of any radical initiator, they occur through the detachment of hydrogen atoms from the collagen macromolecules by the ethyl and ethoxyl radicals formed when oxygen residues oxidize TEB in the reaction medium (Scheme 2) [27].

**Scheme 3:**
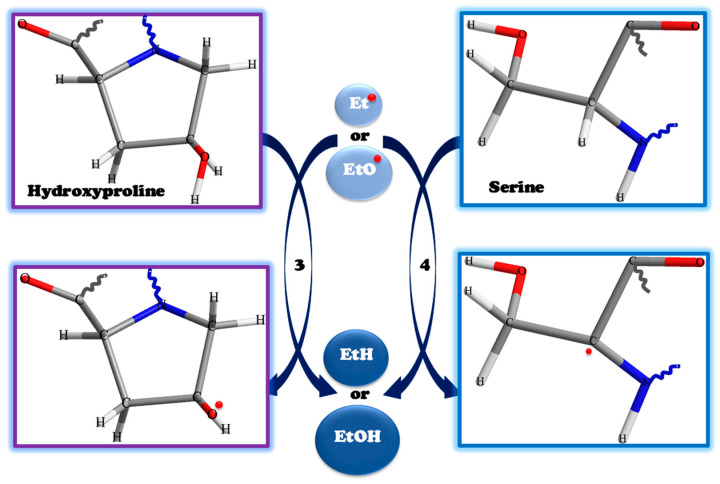
(3) Attack of the hydrogen atom of the hydroxyl group of hydroxyproline. (4) Attack of the hydrogen atom of the hydrocarbon part of serine.

In the amino acid fragments of protein macromolecules, a hydrogen atom is available in the hydroxyl group to attack active radicals (see Scheme 3 for the hydroxyproline example) and in hydrocarbon fragments (see Scheme 3 for the serine example). Collagen is known to contain quite a number of hydroxyl moieties: hydroxyproline (~15%), serine (~4%), hydroxylysine (~1%) [37].

**Scheme 4:**
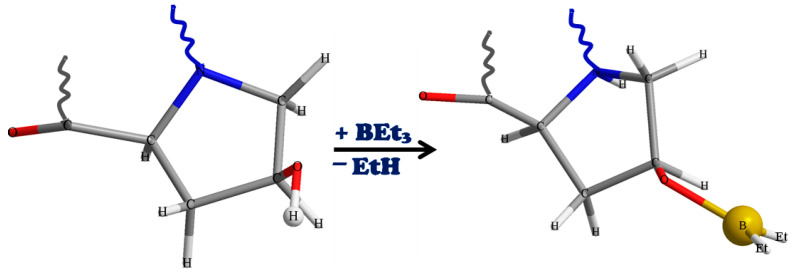
The borination reaction of hydroxyl group by TEB.

**Scheme 5:**
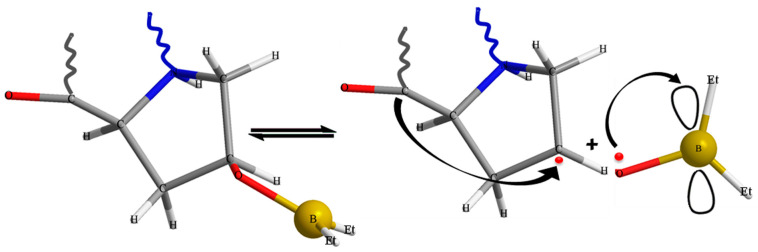
Reversible inhibition reaction.

In addition to these two reactions, the borination reaction of hydroxyl groups is known in the case of trialkylboranes (see Scheme 4 for the hydroxyproline example) [28]. Through this reaction, as in the case of the interaction of the ethoxyl radical produced during triethylboron oxidation (Scheme 2) [28], a labile bond is produced at the end of the polymer chain, thus causing chain growth through the reversible inhibition mechanism (Scheme 5). PBA-PVBE copolymer products produced according to Scheme 5 are described in studies [38].

The contribution of the grafted copolymer produced according to Diagram 6 is low at the start of the process because the reversible inhibition reaction rate is much lower compared to polymerization occurring by bimolecular breakage. However, after oxygen residue depletion in the reaction mixture with the generation of active radicals (Et and EtO), the generation and subsequent polymer formation through bimolecular breakage and macromolecular chain formation follows the reversible breakage reaction according to Scheme 5 [38].

As a result of the radical interactions of collagen with the BA, a BA macromolecular radical is produced, and then the vinyl monomer synthetic fragments become grafted onto the collagen, too.

**Figure 2:**
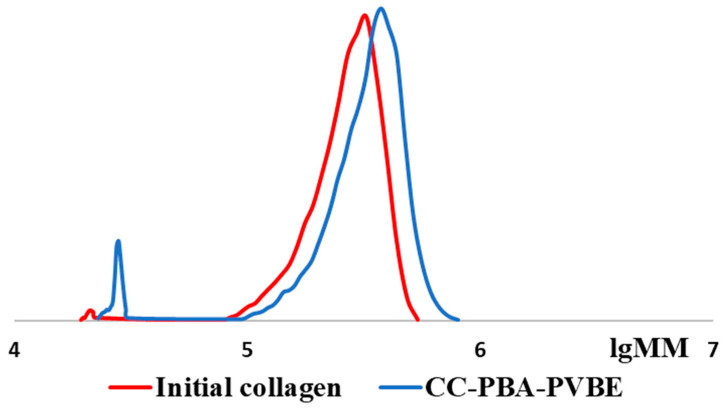
Shift of CC-PBA-PVBE graft copolymer MWD curve compared to initial collagen.

After the synthesis was completed, the aqueous and organic phases were analyzed. Analyses of the aqueous phase of the synthesis confirmed the grafting of the synthetic polymer onto the marine collagen. The GPC method showed an increased graft copolymer molecular weight (MW) (M_w_ = 330 kDa) compared to the original collagen (M_w_ = 280 kDa), while no change of the ratio of the polydispersity value was observed (M_w_/M_n_ = 1.2). Comparison of the molecular weight distribution (MWD) curves of the aqueous solution of the initial collagen and the aqueous phase of the emulsion (Figure 2) also confirmed that grafted copolymer had been produced: the grafted copolymer MWD curve is shifted towards the high-molecular-weight area relative to that of the original collagen, as its MW was increased by the grafted synthetic fragments, although it cannot be excluded that part of the grafted copolymer has a crosslinked structure (due to the chain transfer reaction characteristic of BA and trialkylborane) and does not pass through a syringe filter with a pore diameter of 0.45 microns when preparing samples for GPC analysis.

Similarly, the change of nitrogen content in the sample demonstrated the occurrence of grafting: the amount of nitrogen in the graft copolymer decreased by 20% compared to the original CC. When the dried graft copolymer was washed in chloroform for 10 h in a Soxhlet extractor at 60 °C, no further change in the amount of nitrogen was observed, thus testifying to the chemical bonding of the PBA-PVBE synthetic fragment to the collagen, i.e., graft copolymer formation.

**Figure 3:**
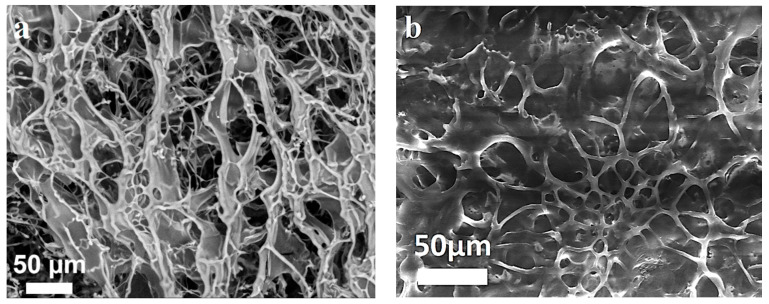
SEM images of initial collagen (**a**) [20] and the CC-PBA-PVBE graft copolymer (**b**).

SEM images were obtained of the morphology and structural organization of both native marine collagen sponge samples and of its graft copolymer obtained in the presence of boralkyl at room temperature (Figure 3). When the SEM images of the sponge samples are compared, a noticeable difference in morphology can be observed, manifested as compaction of the collagen fibers of the structural matrices. In the SEM photograph, they resemble films, as the surfaces of the synthetic fragments have no spatial forms. In addition, the cellular nature of the copolymer with its different sizes of interpenetrating pores is clearly visible, although no copolymer coagulation is caused by the crosslinking formed in the process between collagen macromolecules, making the copolymer different from collagen.

To prove that just the PBA-PBE synthetic fragment was grafted onto the CC under these polymerization conditions, the aqueous phase of the synthesis was subjected to enzymatic hydrolysis using collagenase. As a result of hydrolysis, it was possible to separate the synthetic polymer, which was analyzed by the IR method.

**Figure 4:**
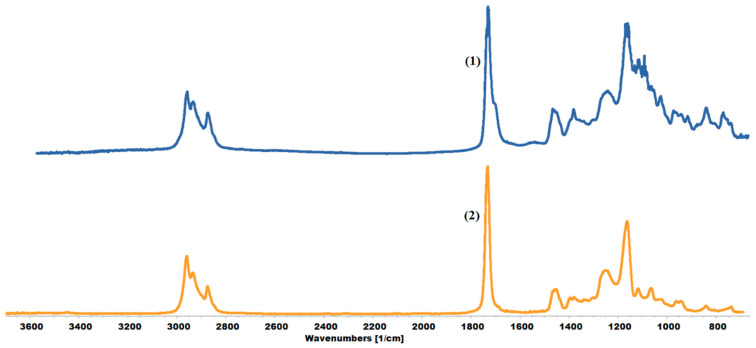
IR spectra of synthetic polymer isolated after hydrolysis of the CC-PBA-PVBE graft copolymer (1) and of the PBA obtained by radical polymerization in the presence of AIBN (2).

See Figure 4 for the IR spectra of the PBA polymer obtained using radical polymerization on azobisisobutyric acid dinitrile (AIBN) and of the polymer isolated after enzymatic hydrolysis of the CC-PBA-PVBE copolymer. Unfortunately, it was not possible to obtain information on the presence of AIBN fragments in the copolymer. An intense band in the 1150–1060 cm^−1^ region is present in both spectra. It is more intense in the case of the synthetic polymer after hydrolysis of the CC-PBA-PVBE, but carbon skeleton vibration bands of average intensity fall in the same region [39]. In this connection, no unambiguous conclusion is possible on the composition of the synthetic fragment.

Analysis of the copolymer solution in chloroform using a calibration graph for the area of the characteristic peak showed that the content of acrylic links in a synthetic polymer isolated from a copolymer with collagen is 51 mol.%. This is strict proof that the synthetic fragment formation contains both BA and VBE fragments. It is especially interesting that the synthetic fragment has alternating structures of BA and VBE fragments. This follows from the known data on the inability of the VBE monomer to form a homopolymer during radical initiation, as well as previously obtained data on the structure of the BA-VBE copolymer [38].

**Figure 5:**
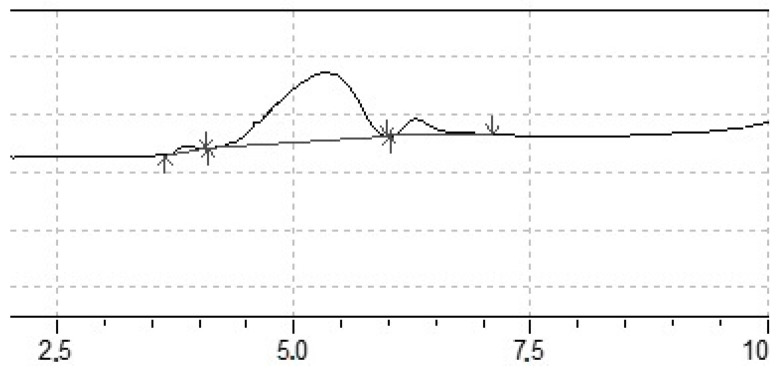
Chromatogram view of the CC-PBA-PVBE copolymer synthetic fragment.

In addition, the GPC method showed that the synthetic polymer isolated after collagenase hydrolysis yields a multimodal spectrum with different MW values (Figure 5). It was not possible to calculate the actual MW values, as no conversion coefficients were available from PMMA standards for PBA-PVBE in tetrahydrofuran at 40 °C.

**Figure 6:**
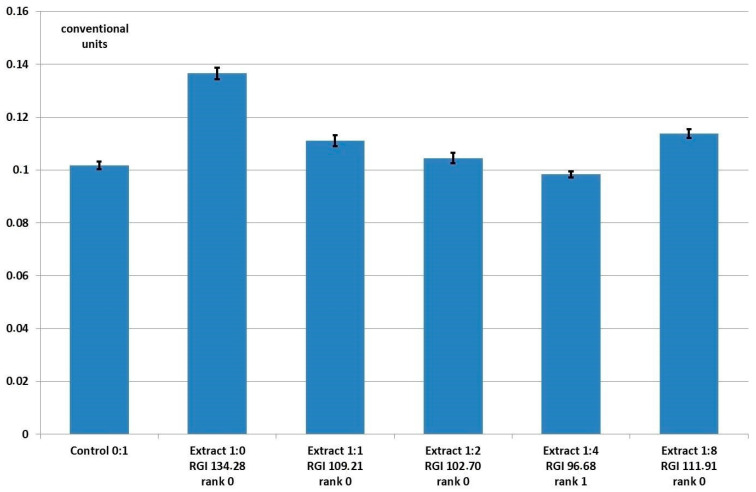
Cytotoxicity evaluation results of CC-PBA-PVBE copolymer in aqueous solution (optical density—OD, wavelength 540 nm).

As noted earlier, their cytotoxicity is an important criterion for polymers intended for regenerative medicine. The MTT assay results showed zero evident cytotoxicity during studies on a sample of this new copolymer when tested in a separated aqueous phase emulsion. However, this promising result was overshadowed by more detailed studies. In particular, the optical density values in both the control and sample wells were extremely low (Figure 6). This indicated only a small number of viable cells, and microscopic examination showed that the cells had suffered the effects of toxicity.

**Figure 7:**
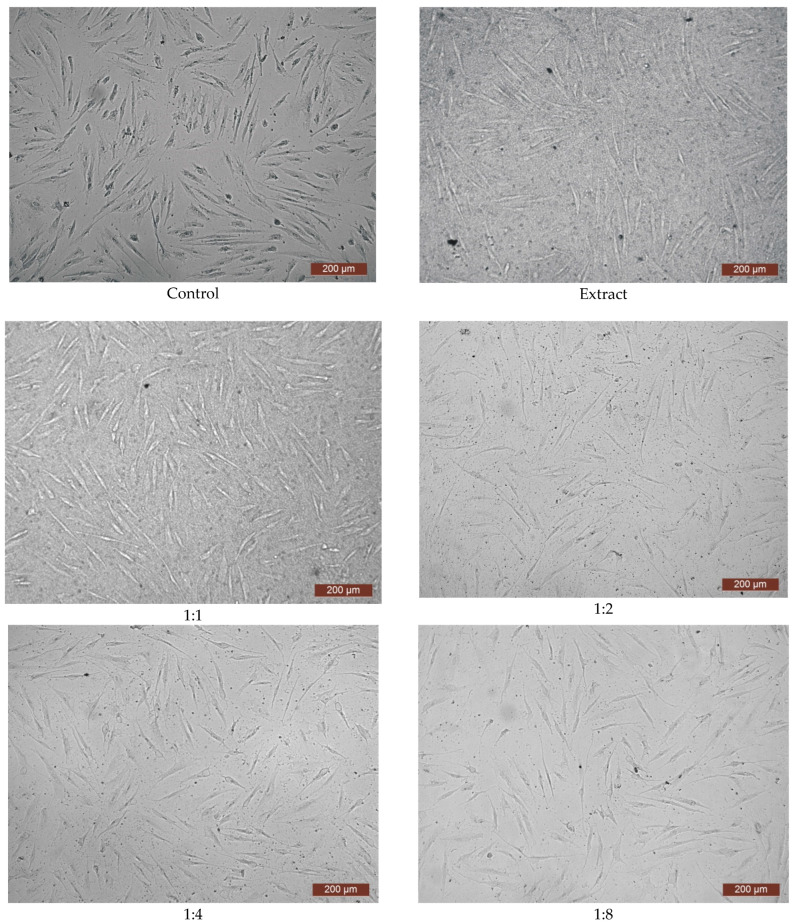
Representative pictures of microscopic images during the interaction of an HDF culture with aqueous solutions of the CC-PBA-PVBE copolymer sample.

**Figure 8:**
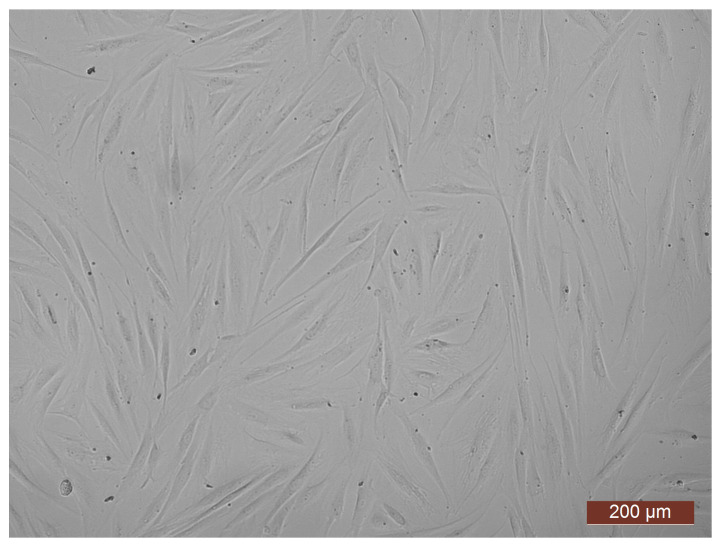
HDFs—the state of the cell culture before the MTT test.

The cell morphology was clearly disturbed—many cell contours were discontinuous and jagged, while other cells had become spherical (Figure 7). This pattern significantly differed from the state of the cells before the MTT test—the HDFs had a typical morphological pattern (spindle-like shape with even contours) and formed a subconfluent monolayer (Figure 8). It appears that the sample contained minor admixtures of residual monomers that had evident toxicity [40,41,42]. During the MTT assay, the control wells were located on the same plate in close proximity to the wells with the test samples. The close proximity of the control and test samples on the assay plate probably caused the toxicity seen in the control cells. The latter was accompanied by a decrease in the OD of both the control and test samples. Considering that evaluation of the level of cytotoxicity during the MTT assay is based on the OD ratios of the control and test samples (see Section 2.8), toxification of the control cells resulted in there being no difference between the control and test samples. On this basis, the results of the MTT assay cannot be considered valid. Thus, the cytotoxic effect can be judged only from the microscopic images of the cells.

**Figure 9:**
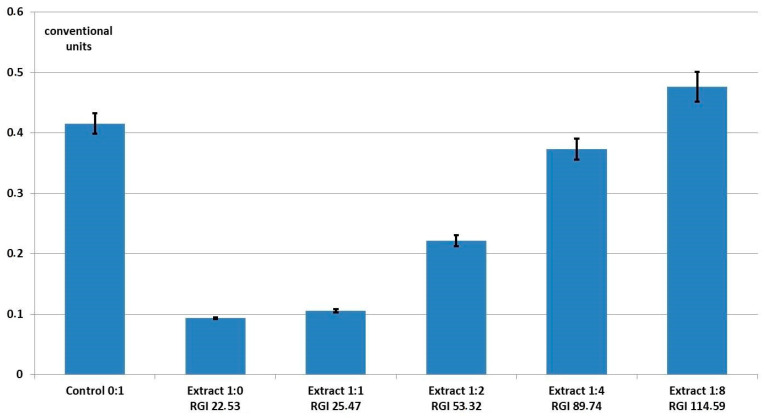
Cytotoxicity evaluation results of a dried sample of the CC-PBA-PVBE copolymer (optical density—OD, wavelength 540 nm).

To reveal the true level of cytotoxicity of the grafted copolymer sample, an aqueous solution of it was dried in a Petri dish to a constant weight. Then, the sample was placed into cell culture medium, incubated for 24 h, and the resulting extract was tested as before. According to its MTT assay results, this sample extract corresponded to cytotoxicity of grade 4 (evident cytotoxicity) (Figure 9).

**Figure 10:**
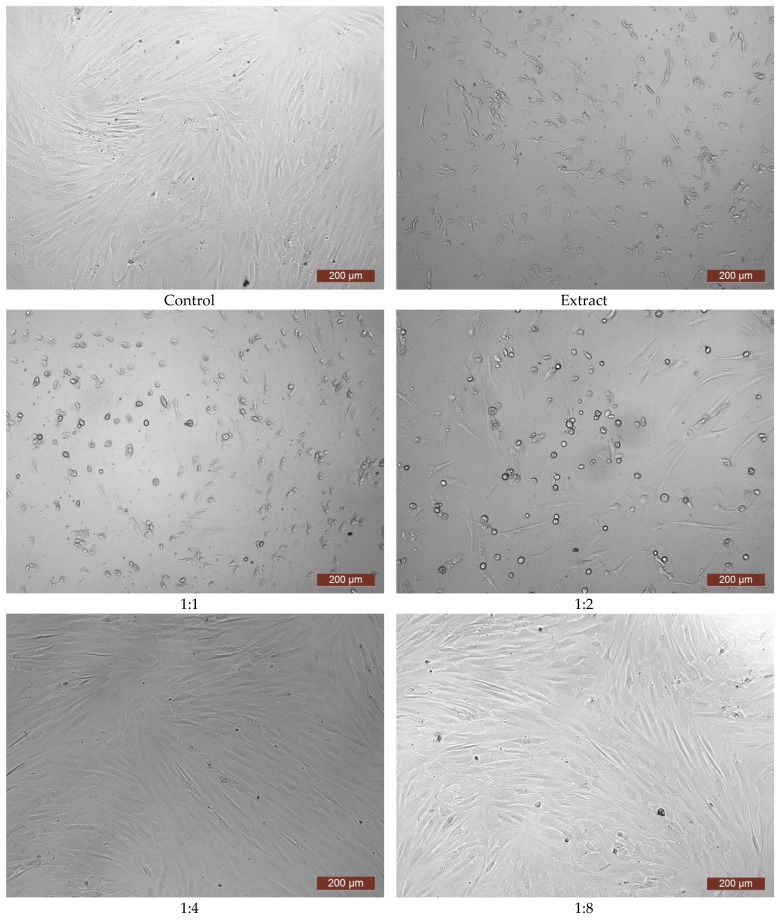
Representative pictures of microscopic images during interaction of HDF cultures with extracts made from a dried sample of the CC-PBA-PVBE copolymer.

The microscopic images corresponded to the MTT assay results. Thus, cells in the presence of the extract showed toxic effects: while some cells had become spherical, others were strongly vacuolated and had indistinct, “jagged” edges (Figure 10). The extract at a 1:1 dilution demonstrated grade 3 cytotoxicity. A multitude of shriveled cells could be visualized within the microscope field of view. At subsequent dilutions, the cytotoxicity grade decreased. Thus, when the extract was diluted to 1:2, moderate cytotoxicity corresponding to grade 2 was observed. Some HDF cells with characteristic morphology could be seen within the microscope field of view, along with a significant number of spherical cells. Dilution to 1:4 corresponded to cytotoxicity grade 1, and dilution to 1:8 demonstrated cytotoxicity grade 0. Here, the HDFs formed a subconfluent monolayer and were virtually indistinguishable, visually, from the controls. This allows us to state that a concentrated extract of the new copolymer is cytotoxic, but that this toxicity is effectively completely lost when its concentration is reduced. The latter indicates that diluted copolymer solutions could be used to obtain materials for regenerative medicine.

## 4. Conclusions

Synthesis was performed, and the resulting properties were investigated for a PBA-PVBE synthetic copolymer (synthesized using trialkylborane) grafted onto cod collagen. The structure, composition, and properties of the new graft copolymer were established using physical and chemical analytic methods: gel permeation chromatography (GPC), scanning electron microscopy (SEM), elemental analysis (CHNS), IR spectroscopy. Further information was provided by hydrolysis of the CC-PBA-PVBE graft copolymer using collagenase, followed by analysis of the isolated, previously grafted synthetic fragment using GPC and infrared (FTIR) spectroscopy. This established that the grafted polymer contained BA and VBE fragments. Evaluation of the cytotoxicity of the grafted copolymer using the MTT assay method indicated the possibility of using diluted copolymer solutions to obtain materials for regenerative medicine. In further studies, it is planned to study graft copolymers as the basis for scaffolds and to obtain three-dimensional structures by introducing additional components with certain characteristics, such as polyethylene glycol and crosslinking agents of various nature. With modification, the concentration of the peptide substrate will decrease markedly. In addition, it is known that PEGylation always leads to a decrease in the toxicity of samples.

## Data Availability

The data that support the findings of this study are available on request from the corresponding author.
